# Deficit irrigation and planting patterns strategies to improve maize yield and water productivity at different plant densities in semi-arid regions

**DOI:** 10.1038/s41598-017-14133-1

**Published:** 2017-10-24

**Authors:** Qianmin Jia, Lefeng Sun, Shahzad Ali, Donghua Liu, Yan Zhang, Xiaolong Ren, Peng Zhang, Zhikuan Jia

**Affiliations:** 1 Chinese Institute of Water-Saving Agriculture, Northwest A&F University, Yangling, Shaanxi 712100 China; 2 Key Laboratory of Crop Physi-Ecology and Tillage Science in Northwestern Loess Plateau, Ministry of Agriculture, Northwest A&F University, Yangling, 712100 Shaanxi China

## Abstract

Field research was done in two consecutive years to optimize deficit irrigation under different crop densities (low, medium, and high) using the ridge and furrow rainfall harvesting (RFRH) system. We demonstrate that applying deficit irrigation (375 m^3^ ha^−1^) at the flowering stage of maize grown at medium density (M: 75000 plant ha^−1^) under the RFRH system (MIF) can improve soil water storage (0–200 cm) at the bell, filling and flowering stages. MIF increased biomass by 10% and grain yield by 21%, thereby achieving a 17% increase in water use efficiency (WUE) and a 22% increase in precipitation use efficiency (PUE) compared with conventional flat planting (CK_M_). MIF also improved irrigation water use efficiency (IWUE) (9%) and irrigation water productivity (IWP) (46%) compared with no-irrigation under the RFRH system (MI_0_). We observed that applying deficit irrigation (750 m^3^ ha^−1^) at the bell and flowering stage (IBF) had positive effects on dry matter, leaf area, and evapotranspiration, but there were no significant increases in IWUE, IWP, WUE, biomass and grain yield compared with maize grown under IF at low, medium and high plant densities. The average net profit over the two years was 34% higher for MIF compared with the CK_M_ treatment.

## Introduction

Irrigation water supplies are declining in many semi-arid regions of world. Some of the reasons for this decline include extended drought periods, uneven rainfall distribution and decline in groundwater levels^1^. China ranks sixth among countries in total water resources, but water availability per capita is only one quarter of the world average. Due to the lack of surface water, groundwater has become a major source of irrigation in semi-arid regions. Groundwater levels are persistently declining, and over-utilization of these limited water resources has lead to a need to develop water-saving methods to improve farm production and irrigation water use efficiency (IWUE)^2,3^. Therefore, to improve the management and consumption of soil water, enhancing the irrigation water productivity (IWP) of crops grown in semi-arid regions, such as maize, is needed^4,5^.

One strategy that is widely used to increase IWP in arid and semi-arid regions is the furrow irrigation system^6,7^. The ridge and furrow rainwater harvesting (RFRH) system includes a ridge covered with plastic mulching and a furrow without mulching where plants are^8^. The plastic mulch on the ridge collects rainwater and directs it into the furrow, allowing the enrichment of rainwater for crop use^9,10^. Ridges covered with plastic film mulching can conserve rainwater, decrease evaporation and improve the WUE of crops^11,12^. Due to these benefits, as well as the fact that it also increases soil temperature, plastic film mulching is widely used in dry-land farming systems^13,14^. The technique of rainwater harvesting is the most popular technique for rainwater harvesting, conservation, utilization, and results in more efficient use of rainfall, which significantly improves IWP^3,4,6^. However, increasing the amount of irrigation to improve maize production is not a practical option in semi-arid regions of northern China. Furthermore, full irrigation leads to higher evapotranspiration (ET) and biomass, but it does not produce the highest grain yield and decreases WUE and irrigation water productivity (IWP)^15^. Thus, the main goal for improving maize yield under deficit irrigation is the development of water-saving cultivation strategies. For a long time, many scientists have focused on optimizing irrigation method and irrigation scheduling, and deficit irrigation has been greatly promoted in recent years. The timing of irrigation has a large impact on crop dry matter. For example, in maize it is critical to irrigate at the appropriate time to minimize stress during the milk and dough growth stages^16^. Although there have been many studies on water-saving agricultural practices, little information is available on the effect of reducing irrigation by fully utilizing the available rainfall under a RFRH system on crops grown under different plant densities.

Maize is particularly susceptible to inadequate irrigation due to its high water consumption^17^. If water is limited, it is important to know at which growth stages to supply deficit irrigation to achieve optimal yields, IWP and net profit^18^. For arid and semi-arid regions, crop yield under limited irrigation depends on the amount of accessible water, which consists of rainfall and available soil moisture, and the timing of water availability^15,16^. The amount of irrigation is also important; excessive irrigation can reduce IWUE, whereas limited irrigation may result in higher IWP and IWUE and reduced ET at field scales^19^. Hence, the effect of deficit irrigation on maize yield and WUE is strongly influenced by soil moisture and irrigation patterns^15^.

In recent years, increasing planting density has also been found to be a simple and effective method for increasing maize yield in semi-arid regions^20^. For example, Lamm *et al*.^19^ showed that higher maize planting densities produce maximum yields. Increasing plant density usually increases crop yields until an optimum number of plants per unit area are reached^21^. Under irrigation plant densities from 50,000 to 56,000 ha^−1^ are considered optimal because competition between plants grown at higher densities results in reduced yields^22^. Soil moisture content and soil fertility are positively correlated with plant density^23^. Plant densities under deficit irrigation could possibly be increased because plants are shorter and have decreased numbers of leaves and decreased leaf area when water is limited. Jose *et al*.^24^ showed that with increased planting density, leaf area and biomass increased, and chlorophyll content, plant lodging resistance, grains per ear and thousand grain weights decreased. Al-Kaisi and Yin^21^ reported that the effect of plant density on total dry matter plant^−1^ was mainly observed after the flowering stage. Little information is available about optimal maize planting densities for deficit irrigation when grown under the RFRH system.

Using the RFRH system has the potential to increase maize productivity in semi-arid regions, but how plant density affects crop yield in this system is unknown. Thus, in the present study we examined the combined effects of the RFRH system with deficit irrigation and different plant densities on yield, SWS, WUE, IWP and economic returns with the goals of reducing the amount of irrigation and improving IWUE and IWP. We expect that the use of this practice could support the RFRH system and allow the efficient consumption of precipitation as well as deficit irrigation in arid and semi-arid regions of China and improve water use efficiency.

## Results

### Soil water storage (SWS)

**Figure 1:**
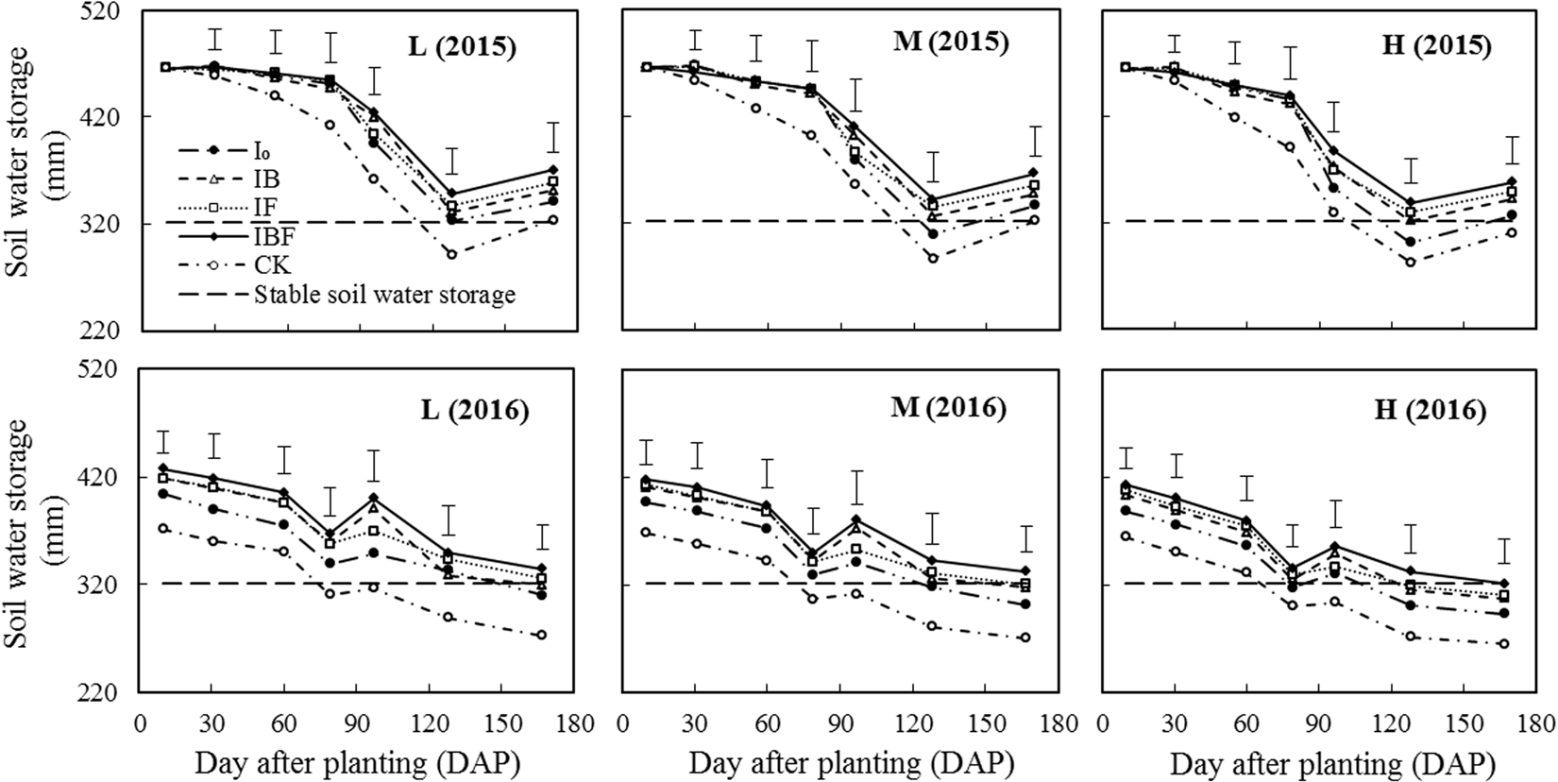
Variation in soil water storage in the 0–200 cm soil layer during maize growth stages under different treatments in 2015 and 2016. L: low plant density (52500 plant ha^−1^); M: medium plant density (75000 plant ha^-1^); H: high plant density (97500plant ha^−1^); I_0_: plastic film ridges with no irrigation; IB: plastic film ridges and irrigation (375 m^3^ ha^−1^) at the bell stage; IF: plastic film ridges and irrigation (375 m^3^ ha^−1^) at the flowering stage; IBF: plastic film ridges and irrigation (750 m^3^ ha^−1^) at the bell and flowering stages; CK: traditional flat planting with plastic film mulching and no irrigation. The Vertical bars represent the value of the least significant difference (LSD) at P < 0.05.

Differences in annual rainfall, evaporation, thermal status and crop water consumption led to obvious variation in SWS (0–200 cm) over the course of maize growth in the RFRH system (Fig. 1). In both years, SWS changed with the amount and distribution of precipitation and with the number of days after planting. Under all treatments in 2015 there were no significant differences in SWS (0–200 cm) from the time of sowing until the jointing stage (0–60 DAP), but SWS decreased from sowing until the filling stage (0–128 DAP). In contrast, under all treatments in 2016, SWS decreased from sowing until the bell stage (0–79 DAP) and increased from the bell until the flowering stage (79–96 DAP) due to the fact that the maximum amount of rainfall (123.3 mm) was received in July. SWS also varied between treatments (Fig. 1). At the bell stage (78 DAP) in 2015, SWS under all RFRH system treatments was significantly higher than under CK at all (low, medium and high) planting densities. However, at the bell stage (79 DAP) in 2016, there was no significant difference in SWS between the I_0_ and CK treatments. In both years, the average SWS at the bell stage under the MIF treatment was 10.5% higher (significant at *P* < 0.05) compared with CK_M_. At the flowering and filling stages and at maturity, SWS under the IF and IBF treatments was significantly higher than under CK at all planting densities. SWS under the CK treatment at the filling stage (128 DAP) was below 321.6 mm (dashed line in Fig. 1) at all planting densities in both years, but SWS under the LIBF, LIF, MIBF and MIF treatments was higher than 321.6 mm. In 2015 from the filling stage to maturity (128–167 DAP), SWS under each treatment increased due to the relatively high amount of rainfall (93.1 mm) received in September, whereas during the same time period in 2016 SWS decreased. SWS under IBF was higher compared with the other treatments at all growth stages at all three planting densities, but was not statistically different from SWS under the IF treatment. The SWS data show that increasing the planting density from low to medium improves SWS stability. With higher planting densities, SWS decreased under same deficit irrigation and planting model.

### Green leaf area and total dry matter

**Figure 2:**
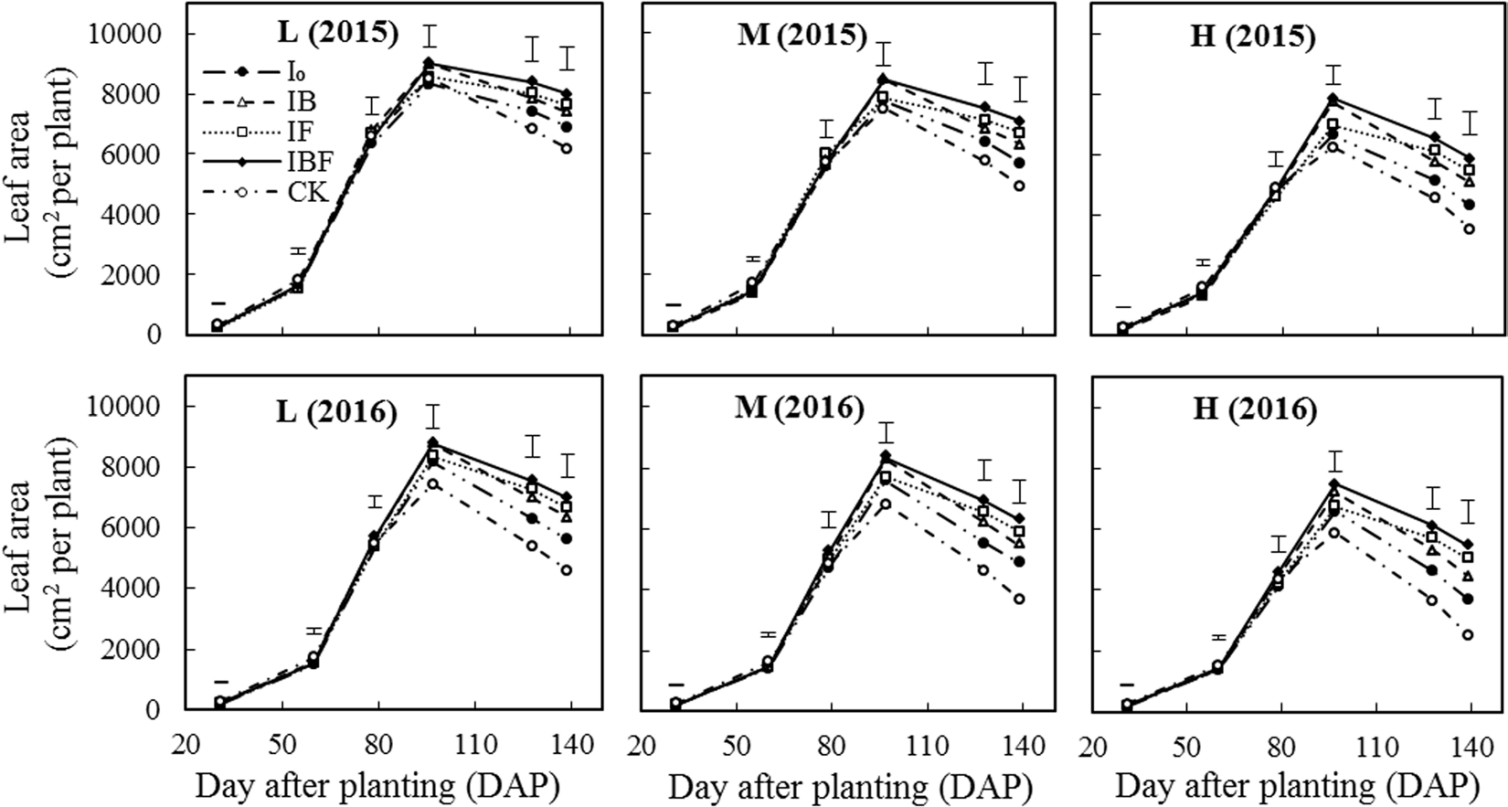
Dynamic changes in green leaf area plant^−1^ during maize growth stages in 2015 and 2016. L: low plant density (52500 plant ha^−1^); M: medium plant density (75000 plant ha^−1^); H: high plant density (97500plant ha^−1^); I_0_: plastic film ridges with no irrigation; IB: plastic film ridges and irrigation (375 m^3^ ha^−1^) at the bell stage; IF: plastic film ridges and irrigation (375 m^3^ ha^−1^) at the flowering stage; IBF: plastic film ridges and irrigation (750 m^3^ ha^−1^) at the bell and flowering stages; CK: traditional flat planting with plastic film mulching and no irrigation. Vertical bars represent the LSD at p = 0.05 (n = 3).

**Figure 3:**
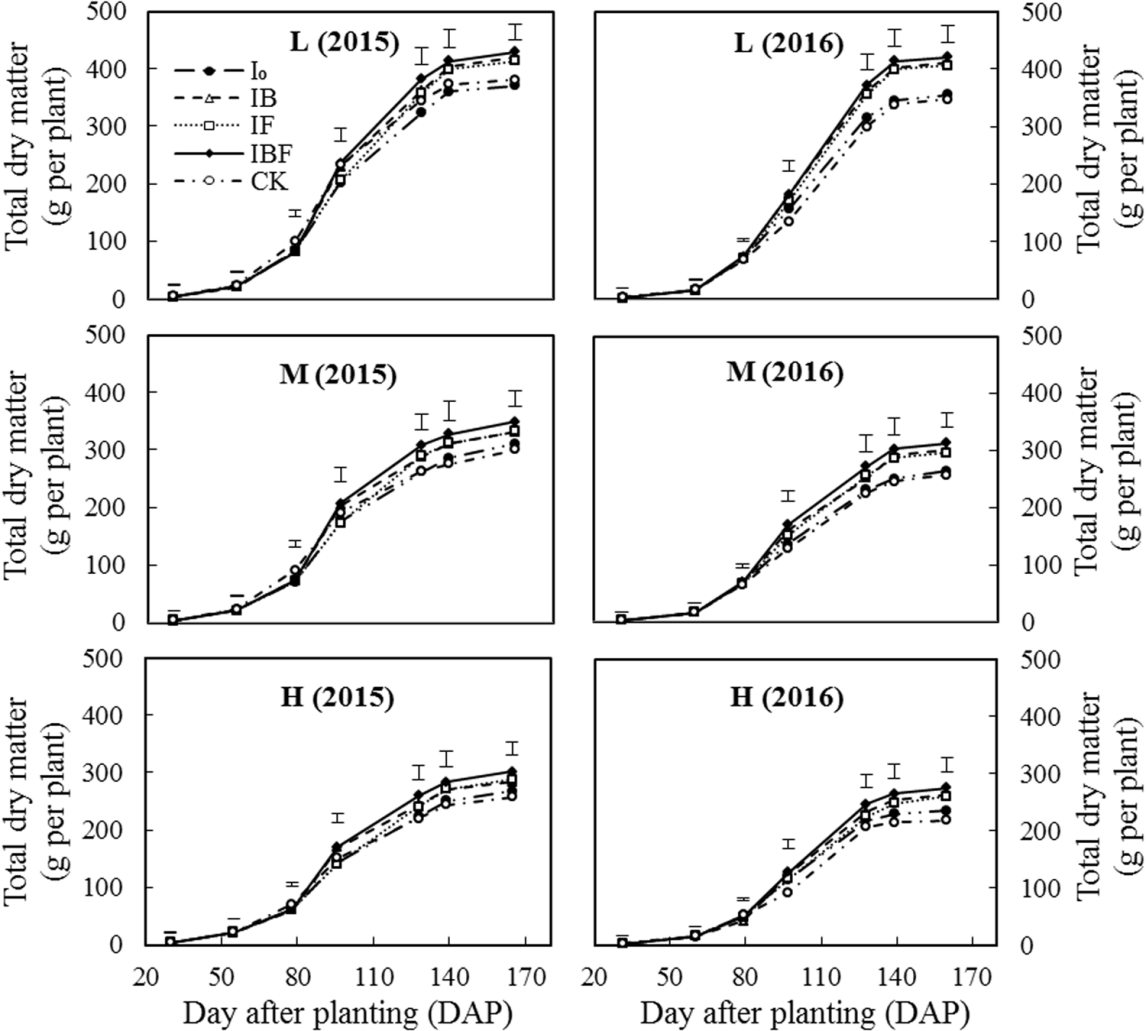
Effect of different treatments on total dry matter (shoot + root) plant^−1^ during maize growth stages in 2015 and 2016. L: low plant density (52500 plant ha^−1^); M: medium plant density (75000 plant ha^−1^); H: high plant density (97500plant ha^−1^); I_0_: plastic film ridges with no irrigation; IB: plastic film ridges and irrigation (375 m^3^ ha^−1^) at the bell stage; IF: plastic film ridges and irrigation (375 m^3^ ha^−1^) at the flowering stage; IBF: plastic film ridges and irrigation (750 m^3^ ha^−1^) at the bell and flowering stages; CK: traditional flat planting with plastic film mulching and no irrigation. Vertical bars represent the LSD at p = 0.05 (n = 3).

The RFRH system combined with different planting densities and timings of deficit irrigation had a significant effect on leaf area and total dry matter (*P* < 0.05, Figs 2 and 3). Under all treatments in both years there were no significant differences in leaf area from the seedling stage until the bell stage (30–79 DAP). The leaf area plant^−1^ increased slowly during the early growth stage (0–60 DAP) before increasing rapidly in the middle growth stage (60–96 DAP), peaking at the flowering stage (96 DAP), and then decreasing gradually in the late growth stage (96–140 DAP). At the filling and dough stages, the leaf area plant^−1^ under the IF and IBF treatments were significantly higher than under CK and I_0_ (*P* < 0.05, Fig. 2) at all three planting densities in both study years, but there was no significant difference between the IF and IBF treatments.

In both 2015 and 2016, total dry matter was significantly affected (*P* < 0.05, Fig. 3) by planting density and deficit irrigation schedule. However, during the early growth stage (30–79 DAP), there were no significant differences in total dry matter plant^−1^ between treatments in both study years. Total dry matter per plant increased slowly during the early growth stage, quickly during the middle growth stage (79–128 DAP), and gradually during the late growth stage (140–167 DAP). At the flowering stage (96 DAP), the total dry matter plant^−1^ under the IBF treatment was not significantly different from CK at all three planting densities in 2015, whereas in 2016 the total dry matter under IBF treatment was significantly higher than under CK. In both study years, maize grown under the IF and IBF treatments produced significantly more total dry matter plant^−1^ compared with maize grown under CK treatment at the dough (140 DAP) maturity stages (167 DAP). However, there was no significant difference in total dry matter between the IF and IBF treatments during all maize growth stages, except for the flowering stage in 2015.

### Biomass, grain yield, and harvest index

In both 2015 and 2016, maize biomass and grain yields in the RFRH system were significantly different compared with conventional flat planting (Table 1). In both years, the ranking of RFRH treatments by largest increase in average biomass and grain yield compared with the CK_L_, CK_M_ and CK_H_ treatments is as follows: MIBF > MIF > HIBF > HIF > LIBF > LIF > HIB > MIB > LIB > HI_0_ > MI_0_ > and LI_0_. The two-year averages indicated that maize biomass yield at low planting density increased significantly (*P* < 0.05) compared with CK_L_, with the yield for the LIBF, LIB, LIF, and LI_0_ treatments increasing by 3.81 t ha^−1^ (21%), 3.30 t ha^−1^ (18%), 3.04 t ha^−1^ (17%), and 0.42 t ha^−1^ (2%), respectively. The biomass yield for maize grown at a medium density increased significantly compared with CK_M_; the yield for maize grown under the MIBF, MIF, MIB, and MI_0_ treatments increased by 4.21 t ha^−1^ (22%), 3.72 t ha^−1^ (19%), 2.97 t ha^−1^ (15%), and 0.55 t ha^−1^ (3%) respectively. The biomass yield for maize grown at a higher density also increased significantly compared with CK_H_; the yield for plants grown under HIBF, HIB, HIF, and HI_0_ increased by 5.58 t ha^−1^ (26%), 4.52 t ha^−1^ (21%), 4.08 t ha^−1^ (19%), and 0.92 t ha^−1^ (4%), respectively.

**Table 1:** Effects of different treatments^abc^ on maize biomass yield (t ha^−1^), grain yield (t ha^−1^), harvest index (HI), evapotranspiration (ET, mm), water use efficiency (WUE, kg ha^−1^ mm^−1^) and precipitation use efficiency (PUE, kg ha^−1^ mm^−1^) in 2015 and 2016^d^.

Year	Treatments	Biomass yield (t ha^−1^)	Grain yield (t ha^−1^)	HI	ET (mm)	WUE (kg ha^−1^ mm^−1^)	PUE (kg ha^−1^ mm^−1^)
2015	LI_0_	19.46 ± 1.05^c^	10.62 ± 0.54^b^	0.54 ± 0.02^a^	457.14 ± 9.91^bc^	23.32 ± 0.44^b^	31.68 ± 1.58^b^
	LIB	22.06 ± 1.04^ab^	11.52 ± 0.59^ab^	0.52 ± 0.03^a^	473.60 ± 17.77^ab^	24.32 ± 0.88^ab^	34.37 ± 1.35^ab^
	LIF	21.73 ± 1.20^ab^	11.96 ± 0.71^a^	0.55 ± 0.03^a^	468.88 ± 20.84^ab^	25.51 ± 1.05^a^	35.68 ± 1.55^a^
	LIBF	22.54 ± 1.60^a^	12.05 ± 0.66^a^	0.53 ± 0.04^a^	488.56 ± 15.39^a^	24.66 ± 1.03^ab^	35.95 ± 1.70^a^
	CK_L_	19.99 ± 0.76^bc^	10.45 ± 0.40^b^	0.53 ± 0.03^a^	448.68 ± 20.76^c^	23.20 ± 0.97^b^	31.18 ± 1.03^b^
	MI_0_	23.60 ± 0.92^b^	11.35 ± 0.60^b^	0.48 ± 0.03^a^	468.04 ± 19.52^c^	24.56 ± 1.36^bc^	33.86 ± 1.71^b^
	MIB	24.79 ± 1.29^ab^	12.21 ± 0.69^ab^	0.49 ± 0.03^a^	486.97 ± 23.79^b^	25.07 ± 1.36^bc^	36.43 ± 2.05^ab^
	MIF	24.92 ± 1.16^ab^	12.99 ± 0.83^a^	0.52 ± 0.04^a^	470.36 ± 23.22^c^	27.62 ± 1.31^a^	38.72 ± 2.05^a^
	MIBF	26.16 ± 1.12^a^	13.11 ± 0.82^a^	0.50 ± 0.02^a^	502.49 ± 26.04^a^	26.10 ± 0.92^ab^	39.11 ± 2.56^a^
	CK_M_	23.26 ± 0.56^b^	11.13 ± 0.58^b^	0.48 ± 0.03^a^	462.25 ± 20.66^c^	23.77 ± 0.64^c^	33.20 ± 1.71^b^
	HI_0_	26.10 ± 0.95^bc^	11.96 ± 0.57^bc^	0.46 ± 0.04^a^	490.44 ± 20.86^b^	24.89 ± 1.18^ab^	35.68 ± 1.47^bc^
	HIB	27.65 ± 1.87^abc^	12.89 ± 0.78^abc^	0.47 ± 0.03^a^	504.04 ± 14.58^a^	25.57 ± 1.60^ab^	38.45 ± 1.96^ab^
	HIF	28.04 ± 1.70^ab^	12.90 ± 0.86^ab^	0.47 ± 0.03^a^	495.61 ± 21.68^ab^	26.03 ± 1.44^a^	38.48 ± 2.16^ab^
	HIBF	29.38 ± 1.31^a^	13.01 ± 0.78^a^	0.47 ± 0.03^a^	519.08 ± 27.02^a^	25.07 ± 1.45^a^	38.81 ± 2.38^a^
	CK_H_	25.05 ± 0.99^c^	11.61 ± 0.71^c^	0.46 ± 0.03^a^	480.89 ± 19.66^b^	23.65 ± 0.53^b^	34.64 ± 1.66^c^
2016	LI_0_	18.67 ± 0.59^b^	9.16 ± 0.34^b^	0.49 ± 0.02^a^	339.64 ± 15.58^c^	26.96 ± 1.10^b^	36.41 ± 1.45^b^
	LIB	21.55 ± 0.91^a^	11.06 ± 0.57^a^	0.51 ± 0.03^a^	390.12 ± 19.83^ab^	28.34 ± 1.12^ab^	43.96 ± 2.07^a^
	LIF	21.29 ± 0.85^a^	11.27 ± 0.57^a^	0.53 ± 0.03^a^	382.01 ± 17.07^b^	29.50 ± 1.52^a^	44.79 ± 2.51^a^
	LIBF	22.06 ± 1.09^a^	11.48 ± 0.47^a^	0.52 ± 0.03^a^	419.96 ± 21.87^a^	27.33 ± 1.07^ab^	45.63 ± 2.73^a^
	CK_L_	18.25 ± 0.55^b^	9.01 ± 0.31^b^	0.49 ± 0.02^a^	338.67 ± 13.70^c^	26.60 ± 1.10^b^	35.81 ± 1.04^b^
	MI_0_	19.78 ± 0.86^b^	9.62 ± 0.53^c^	0.49 ± 0.01^b^	347.09 ± 11.99^c^	27.72 ± 1.02^bc^	38.24 ± 2.00^c^
	MIB	22.20 ± 1.30^a^	11.43 ± 0.65^b^	0.51 ± 0.03^b^	382.18 ± 11.87^b^	29.91 ± 1.67^bc^	45.43 ± 2.84^b^
	MIF	22.95 ± 0.94^a^	12.88 ± 0.66^ab^	0.58 ± 0.03^a^	374.53 ± 18.43^b^	34.39 ± 1.40^a^	51.19 ± 2.30^a^
	MIBF	23.44 ± 0.88^a^	13.05 ± 0.80^a^	0.56 ± 0.04^a^	407.10 ± 21.47^a^	32.06 ± 1.86^b^	51.87 ± 3.25^a^
	CK_M_	19.23 ± 0.47^b^	9.25 ± 0.43^c^	0.48 ± 0.02^b^	347.08 ± 15.42^c^	26.65 ± 1.24^d^	36.76 ± 2.00^c^
	HI_0_	22.19 ± 1.34^b^	9.51 ± 0.47^c^	0.43 ± 0.03^b^	347.15 ± 12.85^c^	27.40 ± 1.59^b^	37.80 ± 2.14^c^
	HIB	25.79 ± 1.86^a^	11.78 ± 0.63^b^	0.46 ± 0.02^ab^	385.71 ± 14.66^b^	30.54 ± 1.41^ab^	46.82 ± 2.28^b^
	HIF	25.35 ± 1.51^a^	12.61 ± 0.58^ab^	0.50 ± 0.03^a^	387.36 ± 16.74^b^	32.58 ± 1.54^a^	50.12 ± 2.99^a^
	HIBF	26.85 ± 1.22^a^	12.91 ± 0.83^a^	0.48 ± 0.03^a^	415.44 ± 19.37^a^	31.08 ± 1.28^a^	51.31 ± 3.15^a^
	CK_H_	21.27 ± 0.87^b^	9.20 ± 0.57^c^	0.43 ± 0.02^b^	346.33 ± 15.41^c^	26.56 ± 1.15^b^	36.57 ± 2.18^c^

^a^L: low plant density (52500 plant ha^−1^); M: medium plant density (75000 plant ha^−1^); H: high plant density (97500 plant ha^-1^); ^b^I_0_: plastic film ridges with no irrigation; IB: plastic film ridges and irrigation (375 m^3^ ha^−1^) at the bell stage; IF: plastic film ridges and irrigation (375 m^3^ ha^−1^) at the flowering stage; IBF: plastic film ridges and irrigation (750 m^3^ ha^−1^) at the bell and flowering stages; ^c^CK_L_: traditional flat planting with plastic film mulching, low plant density (52500 plant ha^−1^) and no irrigation; CK_M_: traditional flat planting with plastic film mulching, medium plant density (75000 plant ha^−1^) and no irrigation; CK_H_: traditional flat planting with plastic film mulching, high plant density (97500 plant ha^−1^) and no irrigation; ^d^Values are given as mean ± s tandard deviation, and different lowercase letters indicate significant differences at P ≤ 0.05 levels in the same line (Duncan’s multiple range test).

The two-year average maize grain yield for the RFRH treatments with a low planting density also increased significantly (*P* < 0.05) compared with the CK_L_ treatment; the mean grain yield of maize grown under the LIBF, LIF, LIB, and LI_0_ treatments increased by 2.03 t ha^−1^ (21%), 1.89 t ha^−1^ (19%), 1.56 t ha^−1^ (16%), and 0.16 t ha^−1^ (2%), respectively. The grain yield of maize grown at a medium plant density increased significantly, with yields for MIBF, MIF, MIB, and MI_0_ increasing by 2.89 t ha^−1^ (28%), 2.75 t ha^−1^ (27%), 1.63 t ha^−1^ (16%), and 0.30 t ha^−1^ (3%) respectively, compared with CK_M_. The grain yield for maize grown at higher densities also increased significantly compared with CK_H_, with yields of plants grown under HIBF, HIF, HIB, and HI_0_ increasing by 2.56 t ha^−1^ (25%), 2.35 t ha^−1^ (23%), 1.93 t ha^−1^ (19%), and 0.33 t ha^−1^ (3%), respectively. During both growing seasons, slightly higher biomass and grain yields were obtained with the IBF treatment compared with the IF and IB treatments under low, medium and high planting densities, but these differences were not significant. These results suggest that by increasing planting density from low to medium, the RFRH system could improve biomass and grain yields. However, growing maize at medium to higher planting densities under deficit irrigation will likely lead to reduced yield.

There were no significant differences in maize harvest index (HI) between treatments at any planting density in 2015 (Table 1). However, in 2016 the HI under the IF and IBF treatments was higher than under the CK treatment under medium and high planting densities, and there was no significant difference between IF and IBF treatment at all three planting densities.

### ET, WUE, PUE, IWUE and IWP

ET was positively correlated with the seasonal rainfall distribution and planting density, and there were obvious differences in ET between treatments (Table 1). At all planting densities and deficit irrigation schedules ET under the RFRH treatments was higher compared with CK. The two-year ET average for the IBF treatment was higher than for the IB and IF treatments. The two-year ET average for low planting density treatments increased significantly (*P* < 0.05) compared with CK_L_; ET for the LIBF, LIB, LIF, and LI_0_ treatments increased by 64.4 mm (17%), 42.0 mm (11%), 35.5 mm (9%), and 8.8 mm (2%), respectively. Seasonal ET under RFRH treatments with a medium plant density increased significantly compared with CK_M_, with ET for MIBF, MIB, MIF, and MI_0_ increasing by 50.2 mm (12%), 30.0 mm (7%), 17.9 mm (4%), and 3.1 mm (1%), respectively. The seasonal ET for high planting density also increased significantly, with HIBF, HIB, HIF, and HI_0_ increasing by 53.9 mm (13%), 31.5 mm (8%), 28.1 mm (7%), and 5.6 mm (1%), respectively, compared with CK_H_.

**Figure 4:**
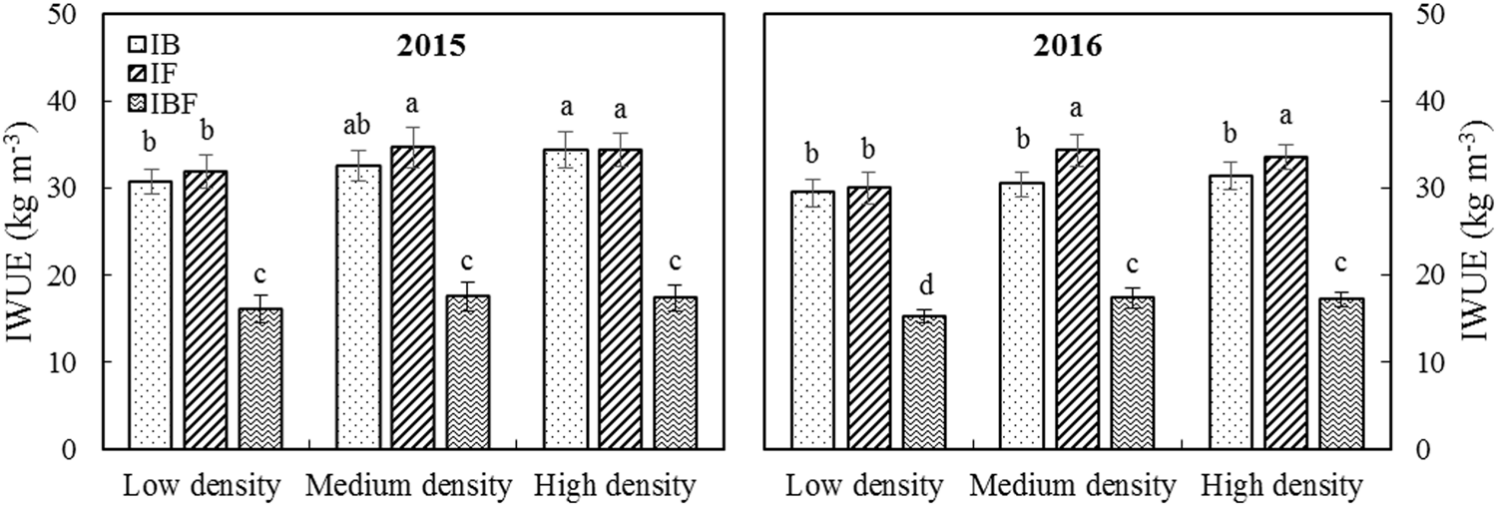
Effects of different treatments on irrigation water use efficiency (IWUE) of maize crops in 2015 and 2016. IB: plastic film ridges and irrigation (375 m^3^ ha^−1^) at the bell stage; IF: plastic film ridges and irrigation (375 m^3^ ha^−1^) at the flowering stage; IBF: plastic film ridges and irrigation (750 m^3^ ha^−1^) at the bell and flowering stages. Different lowercase letters indicate significant differences at P < 0.05, and the error bars represent the standard deviations at the 5% level (Duncan’s multiple range test).

**Figure 5:**
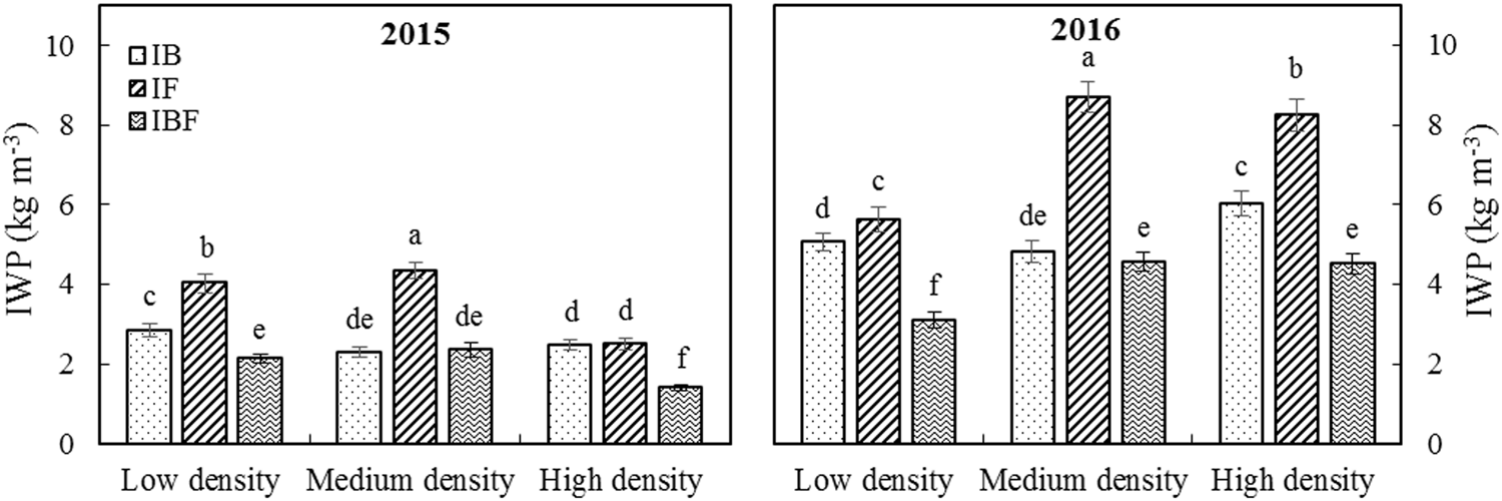
Effect of different treatments on maize irrigation water productivity (IWP) of maize crops in 2015 and 2016. IB: plastic film ridges and irrigation (375 m^3^ ha^−1^) at the bell stage; IF: plastic film ridges and irrigation (375 m^3^ ha^−1^) at the flowering stage; IBF: plastic film ridges and irrigation (750 m^3^ ha^−1^) at the bell and flowering stages. Different lowercase letters indicate significant differences at P < 0.05, and the error bars represent the standard deviations at the 5% level (Duncan’s multiple range test).

The RFRH system at all planting densities and deficit irrigation schedules significantly affected the WUE, PUE, IWUE and IWP of maize crops grown in 2015 and 2016 (Table 1 & Figs 4, 5). The MIF treatment significantly improved WUE, which represents the relationship between water consumption and yield, and PUE compared with CK_M_; under the MIF treatment, the two-year average WUE and PUE were 5.80 kg mm^−1^ ha^−1^ (23%) and 9.97 kg mm^−1^ ha^−1^ (29%) higher, respectively than for the CK_M_ treatment. In addition, the maximum maize yield was achieved under the MIF treatment, in which less deficit irrigation was used (i.e. irrigation only at the flowering stage). The two-year average IWUE for the MIF treatment was 3.87 kg m^−3^ (13%) and 17.06 kg m^−3^ (98%) higher compared with the MIB and MIBF treatments, respectively (Fig. 4). Satisfactory IWP, which indicates the enhancement of maize yield by supplying irrigation, was also obtained for the MIF treatment; IWP was reduced by half compared with the MIBF treatment (Fig. 5). Compared with the MIB and MIBF treatments, the IWP of the MIF treatment was significantly improved by 3.86 kg m^−3^ (80%), and 3.07 kg m^−3^ (89%), respectively. These results suggest that increasing planting density in the RFRH system from low to medium could improve WUE, PUE, IWUE and IWP, whereas increasing planting density from medium to high results would likely lead to a reduction in WUE, PUE and IWUE.

### Economic benefits

The financial benefit was significantly affected by different treatments (Table 2). There were obvious differences in the total costs of the various treatments due to the use of plastic film, labor, cultivation machines, seed for sowing and deficit irrigation amount. However, the IF treatment had a significant positive effect on the net economic profit and output/input ratio for maize grown at medium planting densities. In both years here were significant increases in the net profit of maize grown under the MIF treatments; the net profit increased by 3554 Yuan ha^−1^ (23%) and 6663 Yuan ha^−1^ (45%) in 2015 and 2016, respectively compared with the CK_M_ treatment. Among the different treatments, MIF treatment had the highest output/input ratio in 2015 and 2016 (2.79, and 2.75, respectively).

**Table 2:** Effect of treatments^abc^ on economic benefits (Yuan ha^−1^) of maize grown in 2015 and 2016.

Year	Treatments	Total input (Yuan ha^−1^)	Total output (Yuan ha^−1^)	Net Profit (Yuan ha^−1^)	output/input	Increase over CK (%)
2015	LI_0_	7700	19084	11384	2.48	5
	LIB	8263	21098	12836	2.55	16
	LIF	8263	21787	13525	2.64	20
	LIBF	8825	22013	13188	2.49	18
	CK_L_	8600	19416	10816	2.26	—
	MI_0_	7955	20974	13019	2.64	10
	MIB	8518	22504	13986	2.64	16
	MIF	8518	23796	15278	2.79	23
	MIBF	9080	24121	15041	2.66	22
	CK_M_	8855	20579	11724	2.32	—
	HI_0_	8210	22224	14014	2.71	11
	HIB	8773	23904	15132	2.72	18
	HIF	8773	23960	15187	2.73	18
	HIBF	9335	24280	14945	2.60	17
	CK_H_	9110	21546	12436	2.37	—
2016	LI_0_	7700	16886	9186	2.19	13
	LIB	8263	20289	12026	2.46	33
	LIF	8263	20611	12349	2.49	35
	LIBF	8825	21030	12205	2.38	34
	CK_L_	8600	16601	8001	1.93	—
	MI_0_	7955	17754	9799	2.23	16
	MIB	8518	20965	12448	2.46	34
	MIF	8518	23418	14901	2.75	45
	MIBF	9080	23746	14666	2.62	44
	CK_M_	8855	17093	8238	1.93	—
	HI_0_	8210	17821	9611	2.17	16
	HIB	8773	21895	13122	2.50	38
	HIF	8773	23208	14436	2.65	44
	HIBF	9335	23857	14522	2.56	44
	CK_H_	9110	17215	8105	1.89	—

^a^L: low plant density (52500 plant ha^−1^); M: medium plant density (75000 plant ha^−1^); H: high plant density (97500 plant ha^−1^); ^b^I_0_: plastic film ridges with no irrigation; IB: plastic film ridges and irrigation (375 m^3^ ha^−1^) at the bell stage; IF: plastic film ridges and irrigation (375 m^3^ ha^−1^) at the flowering stage; IBF: plastic film ridges and irrigation (750 m^3^ ha^−1^) at the bell and flowering stages; ^c^CK_L_: traditional flat planting with plastic film mulching, low plant density (52500 plant ha^−1^), and no irrigation; CK_M_: traditional flat planting with plastic film mulching, medium plant density (75000 plant ha^−1^), and no irrigation; CK_H_: traditional flat planting with plastic film mulching, high plant density (97500 plant ha^−1^), and no irrigation; Market Price (Yuan): Maize grain, 1.64 Yuan kg^−1^; biomass, 0.1 Yuan kg^−1^; total input cost = labor + plastic film + machine cultivation + seed for sowing + fertilizers + irrigation water; irrigation water, 1.5 Yuan m^−3^.

## Discussion

Crop productivity in semi-arid regions of China is greatly affected by the amount and distribution of seasonal rainfall; frequent drought in these regions negatively affects crop growth and yields^25,26^. At our study site in Ningxia Province, the annual average rainfall over 11 years is 338 mm, but water evaporation exceeds 1753 mm. Overcoming this imbalance to provide sufficient water is crucial^27^ both for maize production and for the economy in the semi-arid regions of northwest China. Compared with CK, the RFRH system can efficiently maintain a higher SWC during different growth stages of maize, and as a result, promote crop growth and improve WUE and grain yield^28^. In this work, we confirmed that the RFRH system combined with deficit irrigation achieves remarkable improvements in various maize crop parameters. For example, with the IF treatment, SWS, IWUE, IWP, green leaf area, economic benefits and total dry matter accumulation plant^−1^ were increased significantly for maize grown at a medium density compared with maize grown under CK, and the biomass at harvest was increased by 19%.

Previous studies have suggested that by suppressing the evaporation of rainwater, the RFRH technique increases the retention of soil moisture and thus can be used to improve crop water usage, thereby increasing the maize yield, SWS, and PUE^29^. One method to prevent evaporation is to cover the ground with plastic film. Liu *et al*.^30^ found that the soil moisture content of the topsoil in plastic film-mulched plots was 12.2–22.3% higher compared with flat planting plots. In our study, the crop rainwater consumption rate increased during plant growth and development, but the MIF treatment, which imposed only a moderate water stress, ensured the successful vegetative growth of the maize crop. Due to increased rainwater collection and infiltration^25,31^, MIF and MIBF significantly improved the SWS level in the 0–200 cm soil layer at all maize growth stages. SWS in the soil layer from 0–200 cm in depth was higher under the IBF treatment at all growth stages compared with the other treatments at all three planting densities, but was not significantly different from SWS under the IF treatment, even though the deficit irrigation volume was doubled with the IBF treatment. This is because the deficit irrigation water was stored in the planting zone (furrows), and evaporation was inhibited by the plastic mulched ridges^32–34^. Plastic film mulching on ridges and a medium planting density in furrows can reduce evaporation and decrease the total water consumption, thereby positively affecting the maize yield, WUE, and the ET rate^35^.

Another way to prevent water loss is to plant at a higher density. Plants grown at low density consume less water, but there is more evaporation in the field due to the exposure of more ground area to sunlight and windy weather. This is especially the case in semi-arid areas such as the Ningxia province field site where there is a large amount of evaporation. Previous studies have indicated that use of the RFRH system with crops planted at a medium density (65,000 plants ha^−1^) can modify maize water consumption, increase biomass, and convert soil water evaporation into crop transpiration, thereby enhancing the maize production, SWS and WUE^36^. It has been reported that grain yield is positively correlated with ET rate^37^. In our study, the average ET rate over two years for the IBF treatment was higher than for the IB and IF treatments, respectively. Lamm *et al*.^19^ also demonstrated that applying deficit irrigation at the flowering stage under the RFRH system can stabilize the water consumption rate at various stages of maize growth.

Ren *et al*.^34^ confirmed that the optimum precipitation level for ridges covered with plastic film and furrow planting is 230–440 mm and that the enhancement of grain yield was not significant when simulated precipitation exceeded 440 mm. Consistent with this, Griesh and Yakout^22^ found that the use of the RFRH system with a medium (67,000 plants ha^−1^) planting density improved the corn yield by 59–87%, 69–87%, and 21–29% in dry, wet, and heavy rain years, respectively. We also obtained similar results in this study. In 2015 and 2016, the amount of precipitation between April and October was 335.2 mm and 215.6 mm, respectively, and the grain yield under the MIF treatment increased compared with the CK_M_ treatment by 16.7% and 39.2%, respectively. This confirms that the enhancement of maize grain and biomass yields under the RFRH system is better when there is less precipitation. However, the rainy weather of 2015 improved the growth and development of the corn crop and increased ET, leading to a reduction in PUE and WUE in 2015 compared with 2016.

It has been reported that ridges covered with plastic film mulch can improve the soil thermal conditions and moisture content compared with CK^38^ and thereby enhance green leaf area and total dry matter per plant^39^. In addition, Lamm *et al*.^19^ found that planting maize at a density less than 70000 plant ha^−1^ improved green leaf area and total dry matter plant^−1^ during the dry and rainy seasons. In our study, the RFRH system in combination with different planting densities and timing of deficit irrigation had a significant effect on green leaf area plant^−1^ and total dry matter plant^−1^ during all maize growth stages (*P* < 0.05, Figs 4 and 5). The green leaf area plant^−1^ increased slowly during the early growth stage until 60 DAP, before increasing rapidly in the middle growth stage (60–96 DAP), and decreasing gradually in the late growth stage (96–140 DAP). Moreover, the MIF and MIBF treatments had a significant (*P* < 0.05, Fig. 2) effect on green leaf area plant^−1^ in both study years. The total dry matter plant^−1^ after the filling stage (128–167 DAP) under the IBF treatment was significantly higher compared with CK at all planting densities, but no significant difference between IF treatments was observed. This is consistent with results obtained by^21^ who found that the green leaf area and total dry matter per plant were improved when plants were grown at a medium density (67,000 plants ha^−1^) and deficit irrigation was supplied at the flowering stage, whereas there were no significant improvements when irrigation was supplied at both the bell and flowering stages.

When there are shortages of water resources for irrigation, dependence on irrigation could be reduced by consuming local precipitation more effectively^40^. In our study, the MIF treatment significantly improved WUE and PUE compared with the CK_M_ treatment; the two-year average WUE and PUE under the MIF treatment were 5.80 kg mm^−1^ ha^−1^ (23%) and 9.97 kg mm^−1^ ha^−1^ (29%) higher than the CK_M_ treatment, respectively (Table 1). The two-year average IWUE was also higher under the MIF treatment, increasing by 3.87 kg m^−3^ (13%) and 17.06 kg m^−3^ (98%) compared with the MIB and MIBF treatments, respectively (Fig. 4). IWP indicates the capability of enhancing maize grain yield by supplying deficit irrigation. A satisfactory IWP was obtained under the MIF treatment; IWP was reduced by half compared with the MIBF treatment (Fig. 5). Compared with the MIB and MIBF treatments, the IWP of the MIF treatment was significantly improved by 3.86 kg m^−3^ (80%), and 3.07 kg m^−3^ (89%), respectively. These results also suggest that by increasing planting density from low to medium in the RFRH system with deficit irrigation could improve WUE, PUE, IWUE and IWP. However, increasing density from medium to high slightly reduces these parameters. Thus, our study confirms that the application of deficit irrigation is directly associated with amount of rainwater and irrigation timing. Deficit irrigation should therefore be determined in a flexible manner based on crop water utilization and SWS.

Net profit is one of the most important factors when evaluating crop water management strategies in semi-arid regions. The use of the RFRH technique with deficit irrigation requires more investment, but the increased input cost can be balanced by improved maize yields and net profit^41^. We found that growing maize at medium density under the RFRH system and supplying deficit irrigation at the flowering stage had a positive effect on net profit, but excessive deficit irrigation (supplying water at both the bell and flowering stages) led to a misuse of water resources and lower net profits (Table 2). Therefore, the net profit under the MIB and MIBF treatments did not increase significantly compared with the MIF treatment. The net profit under the MIBF treatment was a 23% (3554 Yuan ha^−1^) and 45% (6663 Yuan ha^−1^) higher compared with the CK_M_ treatment in 2015 and 2016, respectively. Our results suggest that the MIF treatment is a suitable RFRH system, and the use of this system may reduce the risk of maize production in semi-arid regions.

## Conclusion

Based on two years of field research, we demonstrate that applying deficit irrigation (375 m^3^ ha^−1^) at the flowering stage (IF) of plants grown under the RFRH system at a medium plant density (M: 75000 plants ha^−1^) (MIF) can improve biomass, grain yield and SWS, and reduce ET, thereby achieving a higher WUE, IWUE, IWP and improved PUE. We observed that applying deficit irrigation at both the bell and flowering stages (IBF) had positive effects on total dry matter, leaf area, and ET, but there were no significant increases in IWUE, IWP, WUE, biomass, and grain yield. In addition, net economic profit under the IBF treatment decreased compared with the IF treatment at both medium and low plant densities. The profit benefits also indicate that greater net profits could be obtained by using the MIF treatment. Therefore, our work suggests that MIF is a suitable planting model for increasing maize productivity in semi-arid regions.

## Materials and Methods

### Study site description

**Figure 6:**
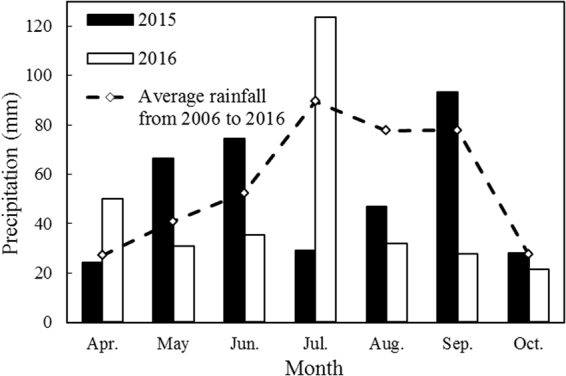
Monthly rainfall in 2015, 2016 and 2006–2016 (11 year average) at Pengyang Experimental Station, Ningxia Province, China.

Field work was carried out during 2015 and 2016 in Pengyang City, Ningxia Province, China. The research site is located at the southern edge of the Ningxia Hui Autonomous Region, at the eastern foot of Liupan Mountain, at a longitude of 106°45′E and latitude of 35°79′N, and at an elevation of 1800 m above sea level. The climatic conditions of the study area are typical of the Loess Plateau with hilly topography, which is characterized as a temperate semi-arid climate with an annual mean evaporation rate of 1735 mm. The annual mean temperature is 6.1 °C, the total duration of sunshine hours is 2518 h yr^−1^. The frost-free period is 140 ~160 days yr^−1^, and the average annual mean rainfall is 410 mm yr^−1^, where over 60% of the rainfall occurs in July–September. The amount of rainfall during the maize growing season was 335.2 mm in 2015 and 251.6 mm in 2016. The monthly rainfall amounts during the two maize growing seasons and the 11-year monthly averages (2006–2016) are shown in (Fig. 6). In the 2015 growing season, the precipitation was well-distributed compared with the 2016 growing season (Fig. 6). The soil at the research site is loess soil with the top soil characterized by a pH of 8.5, a mean bulk density of 1.34 g cm^−3^, and average field water holding capacity and permanent wilting point of 19% and 7.3%, respectively^25^. The stable soil water can be regarded as the lower limit of the effective soil water supply capacity, and its precipitation value varies with different growing seasons. The stable soil water content is 50–75% of the field capacity for loessial soil, and we considered an intermediate value (63% of the holding capacity = 12%) for our comparisons. Thus, the stable soil water storage is 321.6 mm^36^. Characteristics of the soil from 0–60 cm in depth at the research site are shown in Table 3.

**Table 3:** Chemical properties of the top 0–60 cm layer of soil in experimental fields at Pengyang Experimental Station, Ningxia Province, China in 2015 and 2016.

Soil layer (cm)	SOC (g kg^−1^)	SAN (mg kg^−1^)	SAP (mg kg^−1^)	SAK (mg kg^−1^)	STN (g kg^−1^)	Porosity (%)
0–20	9.78	61.52	11.26	168.29	1.01	49.81
20–40	7.99	43.86	7.67	119.36	0.93	47.92
40–60	7.61	40.65	5.85	100.15	0.91	50.19

Note; SOC: soil organic carbon; SAN: soil available nitrogen; SAP: soil available phosphorus; SAK: soil available potassium; STN: soil total nitrogen.

### Experimental design and field management

**Figure 7:**
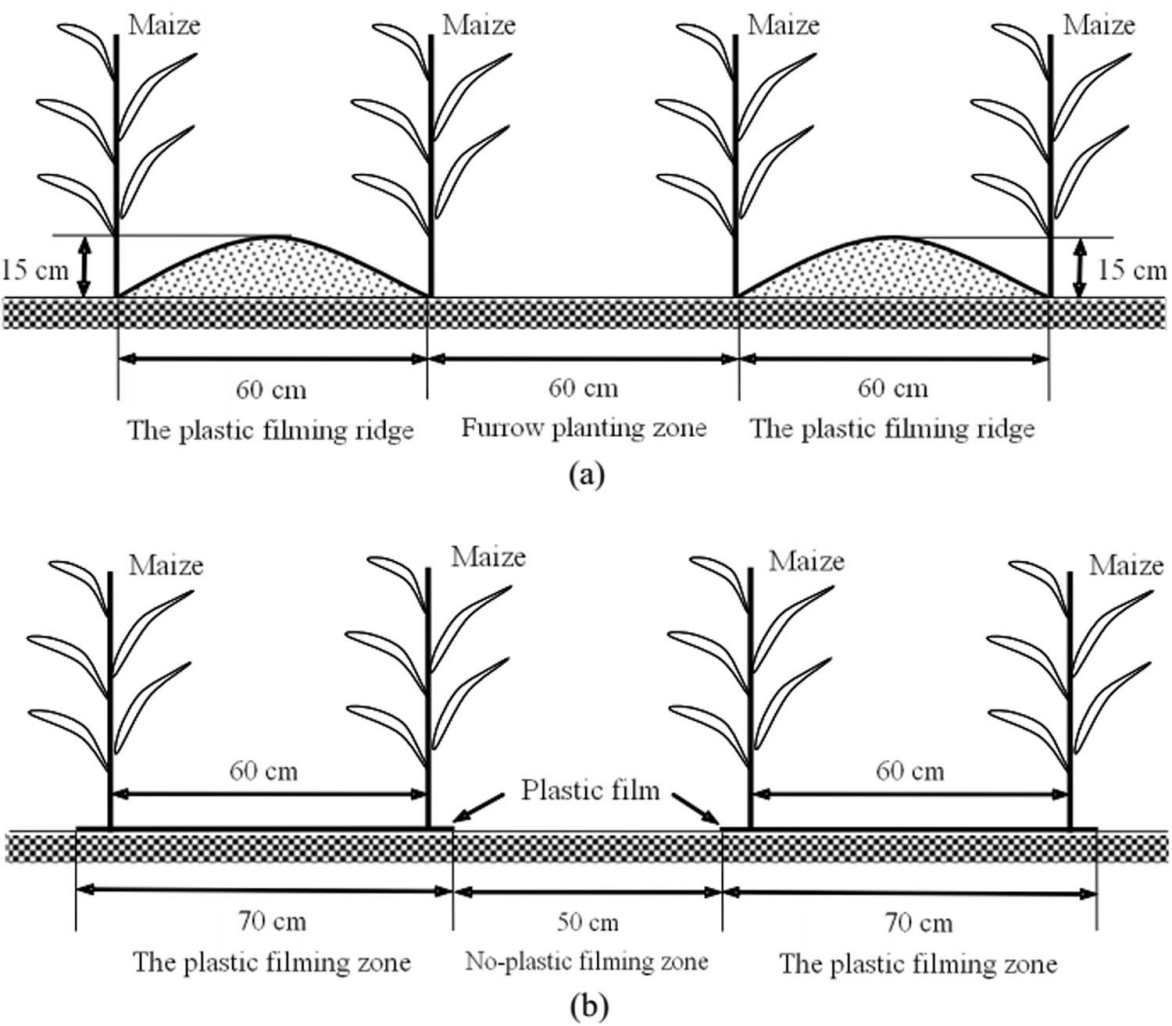
Schematics of the field layouts (**a**) RFRH system (I_0_, IB, IF, IBF treatments); (**b**) conventional flat planting with plastic mulching (CK_L_, CK_M_, CK_H_ treatments). In both systems the effect of deficit irrigation timing and maize planting density was evaluated.

The experiment was performed with a completely randomized block design with three replications. The length and width of each plot was 12.0 m and 4.8 m, respectively (area = 57.6 m^2^). A 1.2 m wide isolation belt was placed between each plot to prevent water leakage. In 2015–16, we conducted field research in semi-arid regions of China to evaluate the effects of five deficit irrigation patterns using the RFRH system: I_0_: plastic film ridges with no irrigation; IB: plastic film ridges and irrigation (375 m^3^ ha^−1^) at the bell stage; IF: plastic film ridges and irrigation (375 m^3^ ha^−1^) at the flowering stage; IBF: plastic film ridges and irrigation (750 m^3^ ha^−1^) at the bell and flowering stages; CK: conventional flat planting with plastic film mulching and no irrigation (Fig. 7). For each irrigation treatment there were three plant densities [low (L): 52500 plant ha^−1^; medium (M): 75000 plant ha^−1^; high (H): 97500 plant ha^−1^]. Irrigation was applied at the large bell stage (July 11, 2015 and July 9, 2016) and/or at the flowering stage (July 29, 2015 and July 31, 2016) with a precise water meter. The actual irrigation amount was calculated according to the actual irrigation area, i.e., each plot was 57.6 m^2^ and the irrigation amount was 2.16 m^3^. The irrigation area of each furrow in the RFRH system was 7.2 m^2^ (0.6 m × 12 m), and the irrigation amount of each furrow was 0.54m^3^ for the bell and flowering stages. For the IBF treatment the irrigation amount for each furrow was 1.08 m^3^. The traditional flat planting and plastic film-covered with no irrigation (CK) plot was arranged as control plot with different plant densities. In the RFRH system, the ridges were 60 cm wide and 15 cm high, and were covered with plastic film (0.9 m wide and 0.008 mm thick), and the furrows planted with seedlings were 60 cm wide. Seeding spacing was as follows: L (31.8 cm), M (22.2 cm), and H (17.1 cm). An illustration of each planting model is shown in Fig. 7.

Maize (Dafeng 30) was sown on April 23 in 2015 and on April 21 in 2016 using a hole-sowing machine with a seeding depth of 4–5 cm. A base fertilizer containing 150 kg ha^−1^ N, and 150 kg ha^−1^ P_2_O_5_ was spread evenly over the furrow and plowed into the soil layer. Top fertilizer containing 150 kg ha^−1^ N was applied on July 10 in 2015 and on July 8 in 2016 at the large bell stage. The fertilizer application was the same in all treatments. The crops were harvested on October 10, 2015 and October 5, 2016. Weeds were controlled manually in each growing season.

### Measurements, calculations and statistical data analysis

Soil water content in the 0–200 cm soil layer was measured before and during sowing and during the maize growth period using a soil drill^34^. A soil sample was taken every 10 cm from the 0 to 20 cm soil layer, and a soil sample was taken every 20 cm from the 20 to 200 cm soil layer. The soil samples were obtained randomly using a soil drill at three locations in the middle of the plant furrow for each treatment. After obtaining the wet weight, the soil samples were then dried for 48 hours in an oven at 105 °C to until a constant weight was reached. The soil water content was then calculated.

Soil water consumption was calculated according to the water balance formula^42^:

1 $${\rm{ET}}={\rm{P}}+{\rm{I}}+{\rm{C}}+({{\rm{SWS}}}_{1}-{{\rm{SWS}}}_{2})-{\rm{D}}-{\rm{R}}$$

where ET (mm) is the evapotranspiration; P (mm) is the total precipitation in the maize growing season; I (mm) is the total irrigation, with I = 0 for the rain-fed treatment; C is the upward flow into the root zone; SWS_1_ (mm) is the soil water storage at planting and SWS_2_ is the soil water storage at harvest, where both SWS_1_ and SWS_2_ in the RFRH treatments represent the mean soil water storage in the middle of the furrow and ridge; D is the amount of water discharged outside the root; and R is the surface runoff. In the experiment, the groundwater level is about 80 m deep, so the groundwater flow to the roots can be ignored. Runoff was never observed as the experimental field was flat, and the drainage was assumed to be insignificant over a 200 cm depth.

Soil water storage was calculated as follows^35^:

2 $$SWS=\sum _{{\rm{i}}}^{{\rm{n}}}{{\rm{h}}}_{{\rm{i}}}\times {{\rm{p}}}_{{\rm{i}}}\times {{\rm{b}}}_{{\rm{i}}}\times 10/100$$

where SWS (mm) is the soil water storage; h_i_ (cm) is the soil layer depth; p_i_ (g cm^−3^) is the soil bulk density of each soil layer; b_i_ is the soil moisture for each soil layer; n is the number of soil layers, i = 10, 20, 40, …, 200.Water use efficiency, irrigation water use efficiency, irrigation water productivity and precipitation use efficiency were calculated using Eqs (3), (4), (5) and (6), respectively^32,43^:

3 $${\rm{WUE}}={\rm{Y}}/{\rm{ET}}$$

4 $${\rm{IWUE}}={\rm{Y}}/{\rm{I}}$$

5 $${\rm{IWP}}=({{\rm{Y}}}_{1}-{{\rm{Y}}}_{2})/{\rm{I}}$$

6 $${\rm{PUE}}={\rm{Y}}/{\rm{P}}$$

where WUE is the water use efficiency relative to the grain yield, Y, in kg ha^−1^ and IWUE is the irrigation water use efficiency. IWP is the irrigation water productivity; Y_1_ (kg ha^−1^) is the yield of irrigated treatment; Y_2_ (kg ha^−1^) is the yield of the CK treatment and I is the amount of deficit irrigation. PUE is the precipitation use efficiency (kg mm^−1^ ha^−1^), Y is the maize grain yield (kg ha^−1^), and P is the amount of precipitation during the whole growth period (mm).

Harvest index based on maize economic yield and biomass yields was calculated as following^44^:

7 $${HI}={Y}_{g}/{Y}_{b}$$

where HI is the harvest index; Y_g_ (kg ha^−1^) is the grain yield, and Y_b_ (kg ha^−1^) is the biomass yield.

Six representative plants were selected to measure the leaf area and dry matter plant^–1^ at 30 days after planting (DAP) and at further intervals of 20–30 days throughout the growing season at each plot. The leaf samples were oven dried at 70 °C for a minimum of 48 h until a constant weight was reached.

At the end of the season, two rows of maize were hand harvested from the middle of each plot with three replicates. The seed and aboveground biomass yields were determined based on a seed water content of 12% for the total land area used, including the combined area of the ridges and furrows.

Net economic profit for each treatment was calculated using the following equations:

8 $${\rm{R}}={\rm{GY}}/{\rm{P}}$$

9 $$\begin{array}{rcl}{\rm{TC}} & = & {\rm{labor}}+{\rm{plastic}}\,{\rm{film}}+{\rm{machine}}\,{\rm{cultivation}}\\  &  & +\,{\rm{seed}}\,{\rm{for}}\,{\rm{sowing}}+{\rm{fertilizers}}+{\rm{irrigation}}\,{\rm{water}}\end{array}$$

10 $${\rm{NEP}}={\rm{R}}-{\rm{TC}}$$

where R is the revenue (Chinese Yuan ha^−1^), GY is the grain yield, P is the local price of maize grain and biomass (1.64 Yuan kg^−1^, 0.1 Yuan kg^−1^), TC is the total cost (Chinese Yuan ha^−1^) and NEP is the net economic profit (Chinese Yuan ha^−1^). All other costs were the same for all treatments.

Data were analyzed by a residual test method before statistical analysis, and the data met the assumption of homogeneity of variances and followed the normal distribution. The experimental data were analyzed with SPSS 13.0 and Excel. Data from each sampling event were analyzed separately. Means among treatments were compared based on the least significant difference test (LSD 0.05).

## References

[1] McGuire, V. L. Water-level Changes in the High Plains Aquifer, predevelopment to 2002, 1980–2002, and 2001–2002. Fact Sheet 2004–3026. U.S. *Geological Survey*, *Lincoln* (2004).

[2] Tan C (2015). Effects of long-term conservation tillage on soil nutrients in sloping fields in regions characterized by water and wind erosion. Sci. Rep..

[3] Zhao H (2014). Ridge-furrow with full plastic film mulching improves water use efficiency and tuber yields of potato in a semiarid rainfed ecosystem. Field Crops Res..

[4] Li XY, Gong JD, Gao QZ, Li FR (2001). Incorporation of ridge and furrow method of rainfall harvesting with mulching for crop production under semiarid conditions. Agric. Water Manage..

[5] Gao Y, Xie Y, Jiang H, Wu B, Niu J (2014). Soil water status and root distribution across the rooting zone in maize with plastic film mulching. Field Crops Res..

[6] Liu XE, Li XG, Hai L, Wang YP, Li FM (2014). How efficient is film fully-mulched ridge–furrow cropping to conserve rainfall in soil at a rainfed site. Field Crops Res..

[7] Li CJ (2016). Towards the highly effective use of precipitation by ridge-furrow with plastic film mulching instead of relying on irrigation resources in a dry semi-humid area. Field Crops Res..

[8] Wang Q (2014). The optimum ridge–furrow ratio and suitable ridge-covering material in rainwater harvesting for oats production in semiarid regions of china. Field Crops Res..

[9] Wang Q (2015). Optimum ridge–furrow ratio and suitable ridge-mulching material for alfalfa production in rainwater harvesting in semi-arid regions of china. Field Crops Res..

[10] Hu Q (2014). Effects of a ridge-furrow micro-field rainwater-harvesting system on potato yield in a semi-arid region. Field Crops Res..

[11] Liu CA (2014). Maize yield and water balance is affected by nitrogen application in a film-mulching ridge–furrow system in a semiarid region of china. Eur. J. of Agron..

[12] Zhang H (2014). Ridge-furrow with full plastic film mulching improves water use efficiency and tuber yields of potato in a-semiarid rain-fed ecosystem. Field Crops Res..

[13] Cao JS, Zhou X, Zhang WJ, Liu CM, Liu ZJ (2012). Effects of mulching on soil temperature and moisture in the rain-fed farmland of summer corn in the Taihang Mountain of China. J. Food Agric Environ..

[14] Han J (2014). Modeling impacts of film mulching on rainfed crop yield in Northern China with DNDC. Field Crops Res..

[15] Kang, S. Z. & Cai, H. J. Theory and Practice of the Controlled Alternate Partial Root Zone Irrigation and Regulated Deficit Irrigation. *China Agricultural Press*, *Beijing* (in Chinese) (2002).

[16] Payero JO, Tarkalson DD, Irmak S, Davison D, Petersen JL (2009). Effect of timing of a deficit-irrigation allocation on corn evapotranspiration, yield, water use efficiency and dry mass. Agric. Water Manage..

[17] Norwood CA (2000). Water use and yield of limited-irrigated and dry-land corn. Soil Sci. Soc. Am. J..

[18] Jose OP, Steven RM, Suat I, David T (2006). Yield response of corn to deficit irrigation in a semiarid climate. Agric. Water Manage..

[19] Lamm FR, Aiken RM, Abou Kheira AA (2008). Corn yield and water use characteristics as affected by tillage, plant density and irrigation. Am. Soci. Agri. and Bio. Eng..

[20] Salah EH, Essam AAL, Mohamed SA, Urs S (2008). Irrigation rate and plant density effects on yield and water use efficiency of drip-irrigated corn. Agric. Water Manage..

[21] Al-Kaisi MM, Yin X (2003). Effects of nitrogen rate, irrigation rate, and plant population on corn yield and water use efficiency. Agron. J..

[22] Griesh, M. H. & Yakout, G. M. Effect of plant population density and nitrogen fertilization on yield and yield components of some white and yellow maize hybrids under drip irrigation system in sandy soil. *In: Proceedings of the International Conference on Plant Nutrition-Food Security and Sustainability of Agro-ecosystems, Madrid, Spain*, pp.810–811 (2001).

[23] Karlen DL, Camp CR (1985). Row spacing, plant population, and water management effects on corn in the Atlantic Coastal Plains. Agron. J..

[24] Jose OP, David DT, Suat I, Don D, James LP (2008). Effect of irrigation amounts applied with subsurface drip irrigation on corn evapotranspiration, yield, water use efficiency, and dry matter production in a semiarid climate. Agric. Water Manage..

[25] Zhang SL, Li PR, Yang XY, Wang ZH, Chen XP (2011). Effects of tillage and plastic mulch on soil water, growth and yield of spring-sown maize. Soil Tillage Res..

[26] Blanco-Moure N, Moret-Fernandez D, Lopez MV (2012). Dynamics of aggregate destabilization by water in soils under long-term conservation tillage in semiarid Spain. Catena..

[27] Grassini P, Yang H, Cassman KG (2009). Limits to maize productivity in Western Corn-Belt: a simulation analysis for fully irrigated and rainfed conditions. Agric. Forest Meteorol..

[28] Gan YT (2013). Ridge-furrow mulching systems an innovative technique for boost-ing crop productivity in semiarid rain-fed environments. Adv. Agron..

[29] Ren X, Peng. Z, Xiaoli C, Jingjing G, Zhikuan J (2016). Effect of different mulches under rainfall concentration system on corn production in the semi-arid areas of the loess plateau. Sci. Rep..

[30] Liu Y (2013). Effects of conservation tillage on grain filling and hormonal changes in wheat under simulated rainfall conditions. Field Crops Res..

[31] Li J (2008). Effects of deep soil desiccation on artificial forestlands in different vegetation zones on the Loess Plateau of China. Acta Ecol. Sin..

[32] Payero JO, Tarkalson DD, Irmak S, Davison D, Petersen JL (2008). Effect of irrigation amounts applied with subsurface drip irrigation on corn evapotranspiration, yield, water use efficiency, and dry matter production in a semiarid climate. Agric. Water Manage..

[33] Peng Z (2015). Effects of straw mulch on soil water and winter wheat production in dry-land farming. Sci. Rep..

[34] Ren XL, Jia ZK, Chen XL (2008). Rainfall concentration for increasing corn production under semiarid climate. Agric. Water Manage..

[35] Qin SH, Zhang JL, Dai HL, Wang D, Li DM (2014). Effect of ridge-furrow and plastic-mulching planting patterns on yield formation and water movement of potato in a semi-arid area. Agric. Water Manage..

[36] Yanhao L (2016). Nutrient and tillage strategies to increase grain yield and water use efficiency in semi-arid areas. Agric. Water Manage..

[37] Jianhua Z (2013). Effects of water deficits on growth, yield and water productivity of drip-irrigated onion (Allium cepa L.) in an arid region of Northwest China. Irrig. Sci..

[38] Zhang, D. Q. *et al*. Effects of plastic film mulching of millet on soil moisture and temperature in semi-arid areas in south Ningxia. *Sci*. *Agric*. *Sin*. **10**, 2069–2075 (In Chinese with English abstract) (2005).

[39] Duan, X. M., Wu, P. T., Bai, X. M. & Feng, H. Micro-rainwater catchments and planting technique of ridge film mulching and furrow seeding of corn in dry-land. *J*. *Soil Water Conserv*. **20**, 143–146 (In Chinese with English abstract) (2006).

[40] Wu Y (2015). Effects of ridge and furrow rainwater harvesting system combined with irrigation on improving water use efficiency of maize (Zea mays L.) in semi-humid area of China. Agric. Water Manage..

[41] Liu CA (2009). Effects of plastic film mulch and tillage on maize productivity and soil parameters. Eur. J. Agron..

[42] Yang, W. Z. & Shao, M. A. Research on soil water of the loess plateau. *Science press*, *Beijin*g (in Chinese) (2000).

[43] Huang YL, Chen LD, Fu BJ, Huang ZL, Gong J (2005). The wheat yields and water-use efficiency in the Loess Plateau: straw mulch and irrigation effects. Agric. Water Manage..

[44] Taisheng D, Shaozhong K, Jingsheng S, Xiying Z, Jianhua Z (2010). An improved water use efficiency of cereals under temporal and spatial deficit irrigation in north China. Agric. Water Manage..

